# Assessment of frailty, daily life activities, and nutrition of elderly immigrants: A household based cross-sectional study

**DOI:** 10.1097/MD.0000000000037729

**Published:** 2024-04-26

**Authors:** Mehmet Sait Değer, Mehmet Akif Sezerol, Muhammed Atak

**Affiliations:** a Department of Public Health, Hitit University – Faculty of Medicine, Çorum, Türkiye; b Department of Public Health, Istanbul Medipol University – School of Medicine, Istanbul, Türkiye; c Department of Public Health, Istanbul University – Istanbul Faculty of Medicine, Istanbul, Türkiye.

**Keywords:** clinical frailty, daily life activities, elderly, immigrant, Katz, mini nutritional assessment, nutrition

## Abstract

With the global migrant population on the rise, it’s imperative to focus on the health status of more vulnerable groups within these communities. The elderly immigrants face myriad physical and psychosocial challenges that significantly impact their health and quality of life. This study aims to investigate the nutrition, daily life activities, and clinical frailty status of elderly immigrants residing in Türkiye. A cross-sectional design was employed in the Sultanbeyli District, focusing on Syrian immigrants aged 65 and over. Participants were surveyed face-to-face by interpreters proficient in Arabic. The questionnaire comprised sociodemographic details, health status, and scales like Katz Daily Life Activities, Clinical Frailty, and Mini Nutritional Assessment. The data analysis was executed using SPSS 22. Continuous variables were presented as mean ± standard deviation (SD) and median, while categorical ones were expressed in numbers and percentages (%). A significance level of *P* < .05 was considered for the analyses. The average age of the participants was determined as 71.64 ± 6.20 years. In the study group, 49.7% were female, 75.5% were younger than 75 years old, 47.7% had less than primary school education, 56.3% were married, 42.4% had a low income level, and 56.9% lived in the same household with 5 or more people. Among the participants in the study group, 47% had walking and balance problems, 29.1% had a history of falls in the last year, 10.6% were disabled, 69.5% complained of pain, 82.8% had a chronic illness, and 43% had polypharmacy. The median value of the KATZ Daily Living Activities scale was 6, the mean score of the Clinical Frailty Score scale was 3.25 ± 1.25, and the mean score of the Mini Nutritional Assessment scale was 12.40 ± 2.15. Among immigrant elderly individuals, 88.1% were able to sustain their lives independently, 13.9% were clinically frail, and 3.3% were at risk of malnutrition. Factors such as age, level of education, socioeconomic status, marital status, number of cohabitants in the household, BMI, neurological problems, walking-balance disorders, disability, and presence of chronic diseases are associated with daily life activities, frailty, and malnutrition status. It is believed that broader field research with greater participation would be beneficial for evaluating the nutritional status of immigrant elderly individuals.

## 1. Introduction

Old age is a progressive phase in human life. Advances in socioeconomic and health sciences worldwide have led to an increase in life expectancy at birth.^[1]^ Consequently, the proportion of the elderly population is gradually rising.^[2,3]^ The global elderly population accounted for 9.5% in 2020, and it is estimated that this figure will double by the year 2050.^[4,5]^

At advanced age, individuals experience significant declines in their physical, cognitive, and social abilities.^[6,7]^ During this period, elderly individuals encounter difficulties in performing daily life activities such as walking, climbing stairs, dressing, and eating due to decreased physical capacities. Additionally, the weakening of skeletal and muscle strength and physiological dysfunction render the elderly more vulnerable to external factors/influences.^[8,9]^ Furthermore, physiological, psychological, and social risk factors associated with aging contribute to inadequate and imbalanced nutrition, in-creasing the risk of malnutrition.^[10]^

Healthy nutrition is a vital aspect for all individuals. Particularly for the elderly, it is a fundamental element for health, autonomy, and quality of life, significantly impacting their aging process.^[11]^ The aging process is intertwined with physiological and psychosocial changes that affect food selection and consumption. This leads to elderly individuals facing inadequate nutrition, a significant public health issue.^[12,13]^ Various physio-logical changes (swallowing difficulties, reduced taste, and appetite), socioeconomic and psychosocial factors, and limited access to health and social services can con-tribute to inadequate nutrition in the elderly.^[14,15]^ Studies have shown that the prevalence of in-adequate nutrition among the elderly ranges from 2% to 8% in the literature and from 20% to 60% in clinical settings.^[14,16–18]^

The decrease in biopsychosocial functional capacity and loss of abilities in the elderly result in a higher incidence of chronic health problems (hypertension, diabetes mellitus, coronary artery disease, chronic obstructive pulmonary disease, stroke, depression), increased dependency on social and psychological support, and the need for the assistance of others.^[19,20]^ Physiological and psychosocial changes in old age, along with resulting issues, impact individuals’ quality of life,^[21]^ increase the risk of illness and death, and impose a significant societal cost from a social and economic perspective.^[1,22,23]^ In the modern concept of healthy aging, it’s not only about protecting individuals from disease and disability but also enabling them to lead long and independent lives by utilizing their physical and mental capacities, and fulfilling social functions.^[6,24,25]^

Immigrants who are forced to leave their country due to reasons like war, disaster, and conflict are one of the disadvantaged groups that should be prioritized in terms of health. Among immigrant groups, elderly immigrants require special attention. The global number of immigrants is increasing. According to the International Organization for Migration (IOM)’s World Migration Report published in 2020, there were estimated to be over 270 million international migrants in 2020, equivalent to about 3.5% of the world’s population.^[26]^ In Türkiye, approximately 3.5 million Syrians primarily reside in border provinces, accounting for 3.73% of the country’s population.^[27]^ Among Syrian migrants, 2.1% fall within the 65 years and older age group.^[28]^

Preserving and enhancing the health status of the community significantly influences the level of welfare in a country. In countries with high welfare levels, the frequency of diseases is lower, access to healthcare is easier, and life expectancy is longer. It is imperative for a country to allocate and efficiently utilize the necessary resources to improve its health and welfare levels. This situation also shapes the country’s migration policy and affects its health status.^[29]^ Various studies have shown that due to the registration system for Syrian migrants in Türkiye, they have insufficient access to healthcare.^[30,31]^ Legal regulations have made it possible for registered migrants to access healthcare services for free. Between 2011 and 2015, the number of applications for healthcare services by Syrians in Türkiye was around 8 million.^[32]^ Preventive healthcare services provided to Syrian migrants (such as immunization, counseling, reproductive health, infant, child, maternal care, screenings, etc.) are partly delivered by Syrian healthcare workers and are provided in Strengthened Migrant Health Centers located in certain provinces. However, primarily, these services are provided by Turkish citizen healthcare workers in primary healthcare facilities.^[32,33]^ Inpatient diagnostic and treatment services are provided in state and university hospitals, which are under the Ministry of Health and staffed by Turkish healthcare workers. Türkiye has the lowest number of physicians and auxiliary healthcare personnel per capita in the European region.^[32]^ This migrant population, in terms of both quantity and quality, also poses a significant economic and physical burden on Türkiye’s healthcare system. Therefore, developing healthcare policies targeting migrants in general and disadvantaged groups such as the elderly specifically, and focusing on healthcare services, is crucial.^[32]^ Additionally, the prevalence of health problems among Syrian migrants in Türkiye and the lack of sufficient research in this area highlight the need for identifying migrants’ healthcare needs and making necessary plans.^[34]^

Elderly immigrants are dealing with common chronic diseases, psychological traumas, and social problems associated with old age. Additionally, they must cope with cultural and economic challenges in adapting to the country they had to migrate to, including language barriers, limited access to healthcare, and psychosocial issues.^[26,35]^ Studies have shown that all these challenges and problems faced by immigrants have a negative impact on their health and quality of life.^[36]^ Therefore, it is essential to implement policies and interventions that mitigate the adverse effects of chronic diseases and psychological and social situations related to migration, allowing elderly immigrants to age healthily and independently.^[37]^ The present study was conducted to examine the daily life activities, clinical frailty, malnutrition status, and related factors of elderly immigrants.

## 2. Materials and methods

### 2.1. Study design and study population

**Figure 1.**
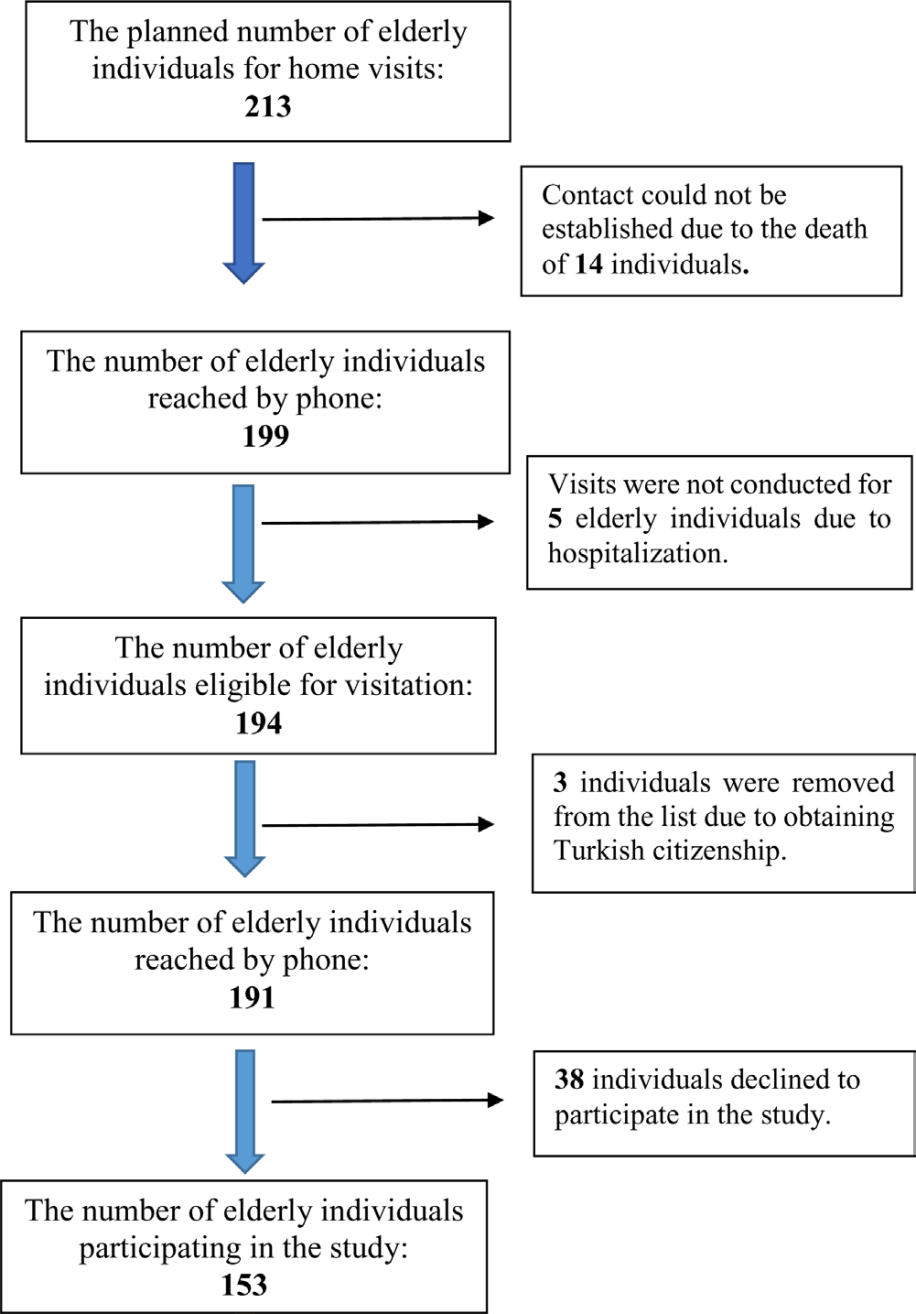
The process of inclusion of participants in the study.

The research was designed as a cross-sectional study. This cross-sectional study was designed to evaluate the health and lifestyle of elderly Syrian immigrants residing in the Sultanbeyli District. Sultanbeyli has a total population of 358,201, of which approximately 6% are Syrian immigrants. Sultanbeyli was chosen as a research location due to being one of the districts with the highest concentration of Syrian immigrants. Additionally, this district is known for having one of the lowest socioeconomic levels in Istanbul. It was planned to visit all of the 213 identified elderly Syrian individuals in the area. However, the actual research was conducted with only 153 of them. Prior to commencing the study, these registered elderly individuals were contacted by phone to establish a visitation schedule (Fig. 1). Subsequently, face-to-face interviews and measurements were conducted at the registered addresses. Measurements and surveys were administered by trained social workers and healthcare professionals. Additionally, Syrian healthcare workers employed at the Sultanbeyli Strengthened Migrant Health Center accompanied the teams conducting home visits as interpreters. With the assistance of Syrian healthcare workers as interpreters, language barriers were overcome, allowing for accurate and effective communication with the participants. Survey questions were conveyed to the participants at a level they could understand, and their responses were confirmed, when necessary, through participant relatives. The study was conducted between January and July 2023.

### 2.2. Measurement tools

In our study, participants were administered a questionnaire consisting of 4 sections, and some measurements were taken. The first section contained statements aimed at obtaining information about sociodemographic characteristics, as well as information related to diseases (hypertension, diabetes mellitus, chronic illnesses, neurological problems, walking-balance impairment) and health status (body mass index, falls, disability, pain, polypharmacy, etc.), which were prepared based on the literature. The other sections included the Katz Activities of Daily Living (ADL) Scale with established validity and reliability studies, Clinical Frailty Score (CFS), and Mini Nutritional Assessment (MNA) Scale.

### 2.3. KATZ activities of daily living scale for the elderly

The KATZ Daily Living Activities Scale is a measure used to assess the extent to which elderly individuals can perform their daily activities independently and to evaluate their self-care functions. The scale evaluates the elderly’s ability to move, dress, eat, bathe, attend to toilet needs, and control urine and stool. The Katz scale consists of 6 items. The status of per-forming each daily function with or without assistance is scored in two options. For functions performed with assistance, 0 points are given, while for functions performed independently, 1 point is given. Scores range from 0 to 6. Scoring low on the scale indicates that the elderly struggle to perform their daily life activities and increasingly rely on others to carry out these functions. Individuals who score 5 to 6 are categorized as “independent, those who score 3 to 4 as “partially dependent, and those who score 2 and below as “totally dependent.”^[7]^

### 2.4. Clinical frailty score (CFS)

The Clinical Frailty Scale is a scale that allows subjective assessment of elderly individuals’ physical activity, cognitive function capacity, and dependency status. The Clinical Frailty Scale enables the detection of frailty in the elderly by evaluating cognitive impairment, comorbidities, and dependency states. The scale score is calculated between 1 and 9 based on clinical assessment. Frailty grading is made with both visual and written explanations at each level. Clinical Frailty score assessment:

1. Very fit: individuals who are robust, fit, and active,
2. Well: individuals with no active disease symptoms but occasionally active,
3. Managing: individuals with medical problems under control but not active except for regular walking,
4. Vulnerable: individuals whose activities are limited due to health problems although not dependent on others in daily life,
5. Mildly frail: individuals who need assistance in complex daily activities (transfer, heavy housework, medication use),
6. Moderately frail: individuals who need assistance in both outdoor and indoor activities,
7. Severely frail: individuals completely dependent on others for physical and cognitive functions but stable in appearance,
8. Very severely frail: completely dependent individuals nearing the end of their lives, where even a mild illness could have a poor outcome,
9. Terminally ill: individuals approaching the end of life with a life expectancy of less than 6 months.

Scoring ≤ 4 on the scale indicates that the individual is not frail, while scoring ≥ 5 indicates frailty.^[38]^ Elderly migrants in our research group were considered “frail” for scores ≥ 5 and “non-frail” for scores < 5.^[9]^

### 2.5. Mini nutritional assessment (MNA)

The “Mini Nutritional Assessment (MNA)” questionnaire was used in our study to describe and assess the nutritional status of elderly immigrants. MNA is a simple and reliable 18-item scale developed to evaluate the nutritional status of the elderly.^[39]^ The scale consists of four sections: general assessment (lifestyle, medication use, physical activity, presence of depression symptoms), subjective assessment (individual perceptions of health and nutrition), anthropometric assessment (BMI, weight loss, and arm and calf circumferences), and short dietary assessment (meal frequency, food and fluid intake, and nutritional autonomy). The scale is divided into two parts, and point calculations are made for each statement. In the first stage, scores range from 0 to 14. Scores of ≥ 12 in the first stage indicate a normal nutritional status, scores of 8 to 11 indicate a risk of malnutrition, and scores of 0 to 7 indicate malnutrition. Individuals with scores ≤ 11 in the first stage undergo the second stage of the scale. Those who complete the second stage can obtain a maximum of 30 points from the scale. In the second stage, scores of ≥ 24 indicate a normal nutritional status, scores of 17 to 23.5 indicate a risk of malnutrition, and scores below 17 indicate malnutrition.^[40]^

### 2.6. Statistical analysis

For statistical analysis, the Katz Activities of Daily Living, Clinical Frailty Score, and Mini Nutritional Assessment scales were considered dependent variables. The Statistical Package for the Social Sciences (SPSS, NY, USA) Program version 22.0 was used for statistical analysis. Continuous variables were expressed as mean ± standard deviation (SD) and median. Categorical variables were presented as numbers and percentages (%). Normality analyses were conducted using the Kolmogorov–Smirnov and Shapiro–Wilk tests, and skewness and kurtosis values of scales with *P* < .05 were examined. Two outlier values were identified and removed, and the analyses were repeated with a total of 151 participants. Values between ± 2 for skewness and kurtosis were considered normally distributed, and parametric tests were applied. Non-parametric tests were applied for values outside the ± 2 range. Chi-square and Fisher’s exact tests were used to compare categorical variables between groups. Student *t* test and One-Way ANOVA test were used for statistical analysis of normally distributed data, while Mann–Whitney *U* test and Kruskal–Wallis tests were used for data that did not show normal distribution. The correlation (Spearman) analysis was performed to assess the relationship between continuous variables. A significance level of *P* < .05 was considered statistically significant.

### 2.7. Ethics approval

Prior to conducting this study, ethical approval was obtained from the Istanbul Medipol University Ethics Committee on November 24, 2022, with protocol number 990. Individuals included in the study were informed about the research and permissions, and they were asked to participate in the study. Our study was conducted in accordance with the Helsinki Declaration and obtained informed consent from all participants.

## 3. Results

A total of 151 Syrian immigrant participants took part in the study. The mean age of the participants was determined as 71.64 ± 6.20 years. The sociodemographic characteristics of the participants included in the study are presented in Table 1.

**Table 1:** Sociodemographic characteristics of participants.

	n	%
Age, years (Mean ± SD)	71.64 ± 6.20	
Age group		
65–74 years (young elderly)	114	75.5
≥75 years (elderly)	37	24.5
Gender		
Female	75	49.7
Male	76	50.3
Education level		
Less than primary school	72	47.7
Primary school	55	36.4
Middle school and above	24	15.9
Marital status		
Married	85	56.3
Not married (single, divorced, or widowed)	66	43.7
Employment status		
Yes	8	5.3
No	143	94.7
Income status		
Low (below minimum wage)	64	42.4
Moderate (minimum wage-poverty line)	82	54.3
High (above poverty line)	2	1.3
Unknown	3	2
Household Size (Mean ± SD)	5.26 ± 2.82	
1–2 people	34	22.5
3–4 people	31	20.5
5–6 people	34	22.5
7 or more people	52	34.4

The mean BMI in the study group was 29.75 ± 6.35 years. In the study group, 75.5% were above normal weight, 70.2% had HT, 24.5% had diabetes mellitus, 47% had gait-balance problems, 82.8% had chronic diseases and 43% had polypharmacy. The data on the health and disease status of the participants are shown in Table 2.

**Table 2:** General health and disease characteristics.

	n	%
BMI (mean ± SD)	29.75 ± 6.35	
BMI classification		
Underweight (≤18.5)	3	2
Normal weight (>18.5–24.9)	34	22.5
Overweight (≥25–29.9)	49	32.5
Obese (≥30)	65	43
Hypertension		
Yes	106	70.2
No	45	29.8
Diabetes mellitus		
Yes	37	24.5
No	114	75.5
Neurological problem		
Yes	15	9.9
No	136	90.1
Smoking status		
Yes	45	29.8
No	106	70.2
Walking and balance disorder		
Yes	71	47
No	80	53
History of fall in the last year		
Yes	44	29.1
No	107	70.9
Disability status		
Yes	16	10.6
No	135	89.4
Pain status		
Yes	105	69.5
No	46	30.5
Chronic disease		
Yes	125	82.8
No	26	17.2
Specific chronic diseases		
Hypertension	89	58.9
Diabetes mellitus	56	37.1
Coronary artery disease (CAD)	35	23.2
Herniated disc	14	9.2
Urological problem	11	7.2
Asthma-bronchitis	10	6.6
Regular medication use (Min: 1, Max: 14)	Mean ± SD: 3.47 ± 3.05	
Less than 4 medications (no polypharmacy)	86	57
4 or more medications (polypharmacy)	65	43

In the study group, 88.1% were able to carry out activities of daily living independently, 86.1% were not clinically frail and 76.2% were in normal nutritional status. Information on scale scores is shown in Table 3.

**Table 3:** Participants’ daily life activities, clinical frailty, and mini nutritional assessment features.

	n	%
KATZ ADL	Median (Min–Max): 6 (0–6)	
KATZ ADL status		
Independent (5–6 points)	133	88.1
Partially dependent (3–4 points)	7	4.6
Dependent (0–2 points)	11	7.3
Clinical frailty score (CFS)	Mean ± SD: 3.25 ± 1.25	
Non–frail (1–4 points)	130	86.1
Frail (5–9 points)	21	13.9
Mini nutritional assessment (MNA) score	Mean ± SD: 12.40 ± 2.15	
Normal nutritional status	115	76.2
At risk of malnutrition	31	20.5
Malnourished	5	3.3

ADL = activities of daily life.

In the KATZ Daily Living Activities Scale, participants under 75 years of age, those with a middle school education or higher, married individuals, and those living with 3 to 4 people in the same household obtained significantly higher scores. In the Clinical Frailty Scale, participants over 75 years of age and those with a primary school education or less obtained significantly higher scores. Similarly, in the Mini Nutritional Assessment scale, participants under 75 years of age obtained significantly higher scores. The scores of the participants according to sociodemographic characteristics in the KATZ Daily Living Activities Scale, Clinical Frailty Scale, and Mini Nutritional Assessment scale are presented in Table 4.

**Table 4:** Comparison of sociodemographic characteristics and health-disease conditions with ADL, CFS, and MNA.

Parameters			ADL	Statistics of test	*P*	CFS	Statistics of test	*P*	MNA	Statistics of test	*P*
	n	%	Mean			Mean ± SD			Mean ± SD		
Gender											
Female	75	49.7	73.65	U = 2673.5	.293	3.33 ± 1.24	*t* = 0.793	.429	12.65 ± 1.67	*t* = 1458	.147
Male	76	50.3	78.35			3.17 ± 1.26			12.14 ± 2.52		
Age group											
65–74 years	114	75.5	80.35	U = 1613	**.001**	3.02 ± 1.05	*t* = −3522	**.001**	12.67 ± 2.00	*t* = 2526	**.015**
≥75 years	37	24.5	62.59			3.97 ± 1.53			11.57 ± 2.38		
Educational level											
Under primary school^1^	72	47.7	68.82	χ^2^ = 10.627	**.005** ^**1-3**^	3.74 ± 1.34	F = 13.538	**.000** ^**1-2**^ ^**1-3**^	12.21 ± 2.00	F = 13.538	.524
Primary School^2^	55	36.4	80.38			2.96 ± 0.92			12.49 ± 2.30		
Secondary and above^3^	24	15.9	87.50			2.46 ± 1.06			12.75 ± 2.25		
Marital status											
Married	85	56.3	81.27	U = 2357	**.007**	3.13 ± 1.23	*t* = −1356	.177	12.41 ± 2.20	*t* = 0.094	.926
Not married	66	43.7	69.21			3.41 ± 1.27			12.38 ± 2.10		
Number of people living with											
1–2 People^1^	34	22.5	81.51	χ^2^ = 8410	**.038** ^**2-3**^ ^**2-4**^	3.26 ± 1.10	F = 2079	.106	12.53 ± 1.87	F = 0.724	.539
3–4 People^2^	31	20.5	85.11			2.77 ± 0.80			12.81 ± 1.62		
5–6 People^3^	34	22.5	71.93			3.47 ± 1.44			12.32 ± 2.19		
≥7 People^4^	52	34.4	69.63			3.38 ± 1.38			12.12 ± 2.54		

ADL = Activities of Daily Living, CFS = Clinical Frailty Score, *F =* One Way ANOVA, MNA = Mini Nutrition Assessment, *t =* Student *t* test, *U =* Mann–Whitney *U* test, χ^2^ *=* Kruskal Wallis test.

1, 2, 3 Statements of groups with significant differences between them.

In the CFS scale, participants who were older than 75 years, had an education level of primary school or lower, had neurological problems and balance issues, and had disabilities, chronic illnesses, and pain obtained significantly higher scores (Table 5).

**Table 5:** Scores of KATZ daily living activities scale, clinical frailty scale, and mini nutritional assessment scale according to health and disease status.

Parameters			ADL	Statistics of test	*P*	CFS	Statistics of test	*P*	MNA	Statistics of test	*P*
Body mass index											
Underweight^1^	3	2	87.5	χ^2^ = 2654	.448	3.33 ± 0.57	*F* = 1023	.384	8 ± 2.64	*F* = 7395	.000 ^1-2^ ^1-3^ ^1-4^ ^2-3^
Normal^2^	34	22.5	75.56			3.44 ± 1.54			11.71 ± 2.82		
Overweight^3^	49	32.5	80.11			3.00 ± 0.97			12.96 ± 1.76		
Obese^4^	65	43	72.60			3.34 ± 1.29			12.54 ± 1.65		
Hypertension											
Yes	106	29.8	70.73	U = 2148	.123	3.23 ± 1.13	*t* = 0.336	.738	12.18 ± 2.41	*t* = −0.762	.449
No	45	70.2	78.24			3.31 ± 1.52			12.49 ± 2.03		
Diabetes mellitus											
	37	24.5	76.22	U = 2083.5	.860	3.30 ± 1.32	*t* = −0.289	.773	12.31 ± 2.20	*t* = −0.954	.343
No	114	75.5	75.31			3.24 ± 1.02			12.68 ± 1.98		
Neurological disorder											
Yes	15	9.9	56.87	U = 733	.004	4.13 ± 1.59	*t* = 2305	.035	10.67 ± 2.49	*t* = −2877	.011
No	136	90.1	78.11			3.15 ± 1.17			12.59 ± 2.03		
Smoking status											
Yes	45	29.8	72.38	U = 2222	.289	3.11 ± 1.40	*t* = −0.838	.405	11.78 ± 2.59	*t* = −2063	.043
No	106	70.2	77.54			3.31 ± 1.19			12.66 ± 1.88		
Walking and balance disorder											
Yes	71	47	63.98	U = 1986.5	.000	3.83 ± 1.44	*t* = 5916	.000	11.68 ± 2.37	*t* = −4001	.000
No	80	53	86.67			2.74 ± 0.75			13.04 ± 1.70		
History of fall in the last year											
Present	44	29.1	67.80	U = 1993	.018	3.30 ± 1.04	*t* = 0.303	.762	12.18 ± 1.95	*t* = −0.833	.407
Absent	107	70.9	79.37			2.23 ± 1.33			12.49 ± 2.22		
Disability status											
Yes	16	10.6	47.94	U = 631	.000	4.56 ± 1.67	*t* = 3420	.003	11.00 ± 2.58	*t* = −2336	.032
No	136	89.4	79.33			3.10 ± 1.10			12.56 ± 2.04		
Pain status											
Yes	105	69.5	72.58	U = 2055.5	.020	3.43 ± 1.24	*t* = 2717	.008	12.09 ± 2.29	*t* = −3152	.002
No	46	30.5	83.82			2.85 ± 1.19			13.11 ± 1.59		
Chronic disease											
Yes	125	82.8	74.94	U = 1493	.298	3.23 ± 1.13	*t* = 2279	.028	12.31 ± 2.13	*t* = 1047	.302
No	26	17.2	81.08			3.26 ± 1.32			12.81 ± 2.20		
Polypharmacy											
Yes (≥4)	65	43	71.42	U = 2497.5	.074	3.48 ± 1.18	*t* = −1956	.052	12.20 ± 2.16	*t* = 0.981	.328
No (<4)	86	57	79.46			3.08 ± 1.28			12.55 ± 2.13		

*F =* One Way ANOVA*, t =* Student *t* test, *U =* Mann–Whitney *U* test, χ^2^ *=* Kruskal Wallis test.

1, 2, 3, 4 Statements of groups with significant differences between them.

In the MNA scale, participants who were younger than 75 years, had a BMI (body mass index) ≤ 18.5, didn’t have neurological problems and balance issues, didn’t have disabilities and pain issues, and didn’t smoke, obtained significantly higher scores (Table 5).

The data showing the relationship between participants and the ADL, CFS, and MNA scales with their sociodemographic characteristics, health conditions, and disease status are presented in Table 5.

In the correlation study of the scales used in the research, a moderate negative correlation was found between KATZ and CFS and a weak positive relationship between CFS and MNA. The data showing the relationship between the scales are shown in Table 6.

**Table 6:** Relationship between KATZ ADL, clinical frailty score and MNA.

		KATZ ADL	CFS	MNA
KATZ ADL	CC	1	**−0.563** **	**−**0.127
	*P*		**.000**	.120
CFS	CC		1	**0.247** **
	*P*			**.02**
MNA	CC			1
	*P*			

** *P <* .05, CC: Spearman’s rho Correlation Coefficient χ^2^.

## 4. Discussion

Factors leading to migration and the challenges of the migration process adversely affect the physical and psychosocial well-being of migrants. Additionally, the presence of age-related risk factors makes immigrant elderly individuals more vulnerable.^[28]^ Furthermore, immigrant elderly individuals living economically, and socially disadvantaged lives should be prioritized for public health interventions.^[41,42]^ The demanding dynamics of the migration process and anxiety contribute to the weakening of migrants’ social skills and self-perception, leading to increased vulnerabilities.^[42,43]^ As the migration process is physically and mentally challenging, generally, healthier and more resilient individuals are more likely to migrate. Additionally, countries that accept migrants as a policy tend to accept healthier migrants. Various studies have shown that both individual and policy contexts providing opportunities for migration for healthier individuals result in migrants being healthier than the native population in the receiving country. This phenomenon, known as the healthy migrant effect, is particularly relevant for adult migrants, although changes in the duration and conditions of life in the receiving country affect this effect and health outcomes.^[44]^ The situation of Syrian migrants in Türkiye, however, is different. Türkiye has embraced an open-door policy, accepting all migrants from Syria since the onset of the civil war in Syria. Additionally, Türkiye’s geographical proximity to Syria has led migrants, particularly, to prefer Türkiye. Due to the open-door policy, it is not feasible to speak of the healthy migrant effect since migrants coming from Syria to Türkiye are not only healthy adults.^[29]^ Therefore, determining the general health status and associated factors of Syrian migrants in Türkiye is crucial for the implementation of health policies. Daily life activities, vulnerability, and malnutrition statuses provide crucial information about the overall health and disease status of migrant elderly individuals. This study aims to determine the factors associated with the daily life activities, clinical vulnerability, and malnutrition statuses of Syrian migrant elderly individuals.

Various conditions significantly influence the health and well-being of migrants. Therefore, sensitive methods considering psychosocial and cultural needs should be used for a comprehensive assessment of migrant elderly groups. In this context, factors such as cultural integration, access to healthcare services, communication problems, health literacy, economic independence, and cultural beliefs should be taken into account.^[45]^ Although Syria’s geographical proximity to Türkiye appears to be a facilitating factor for integration due to cultural similarity, language differences fundamentally complicate the cultural integration of migrants into Türkiye.^[46]^ The language barrier, primarily causing communication problems, hinders the understanding of the healthcare system and access to healthcare services. Syrian migrants have been living in Türkiye for over a decade. Therefore, while language is not a significant problem for those who arrived in Türkiye at a young age or were born and educated in Türkiye, it is a significant barrier to integration, socialization, and access to healthcare services for elderly migrants.^[42,43,47]^ Additionally, distinguishing the effects of pre- and post-migration health risks on their current health status is crucial in evaluating the healthcare services provided to migrants.^[29,42,44,46]^ Therefore, providing strengthened primary healthcare services to disadvantaged groups like migrants is essential to assess their health status, reduce the risk of chronic diseases, and offer comprehensive healthcare.^[48]^

Of the participants in our research group, 75.5% are under the age of 75, 84.1% have primary education or below, 56.3% are married, 96.7% have an income level below the poverty line, and 56.9% live in households with 5 or more people. Participants live in low educational and socioeconomic levels and crowded family environments. Low educational and income levels lead migrants to live and exist in poorer conditions. This situation also negatively affects their ability to afford healthy and balanced nutrition and their pursuit of healthy living behaviors. Additionally, the low educational level of migrants in the research group is associated with insufficient health literacy, resulting in weak health perception among migrants and various problems in accessing and using health services.^[42]^ In Türkiye, factors such as registration procedures, unfamiliarity with the new healthcare system, language barrier, concerns about negative treatment, and cost are prominent barriers to Syrian migrants’ access to and use of health services.^[34,46]^ Various studies have shown that factors such as advanced age,^[23,37]^ low education and socioeconomic level,^[36,37,49]^ limited physical activity,^[23,37,49]^ obesity,^[23,37]^ chronic diseases,^[23,37]^ and inadequate nutrition^[49]^ contribute to poor health outcomes in migrants.

Unhealthy housing and living conditions, physical inactivity, smoking, and inadequate nutrition are well-known risk factors for chronic diseases.^[34]^ Sociocultural beliefs about health can influence migrants’ acquisition, maintenance, and pursuit of healthy living behaviors and their search for healthcare services. On the other hand, negative dietary habits such as excessive salt consumption, low consumption of whole grains and fruits can also predispose to the development of chronic diseases. Obtaining employment opportunities in the country of migration is more challenging for migrants due to reasons such as language problems, low educational level, and lack of profession. The limited employment opportunities lead to low income levels, which in turn can lead to living in crowded family environments.^[46]^ On the other hand, for Syrian migrants who are culturally accustomed to living in crowded family environments, this situation facilitates the functioning of intra-family social support mechanisms. In a study conducted with Syrian migrant elderly people in Türkiye, it was shown that traumatic events, economic uncertainty, and family relationships affect the mental and physical health of the elderly.^[50]^

In the research group, 75.5% of the participants had a BMI ≥ 25, 47% had walking and balance problems, 29.1% had a history of falls in the last year, 82.8% had a chronic disease (Hypertension, Diabetes, Coronary Artery Disease, lumbar hernia, respiratory system diseases), and 43% had polypharmacy (regular use of ≥ 4 drugs per day). The BMI values obtained in migrant elderly people were unexpectedly high. This indicates that migrants need support and guidance for adequate and balanced nutrition. Decreased physical and cognitive capacities of the participants and the presence of chronic diseases can lead to various problems in their daily lives.^[46]^ In addition, the risk of encountering various accidents and chronic pains and the frequency of daily medication use increase.^[51]^ Chronic disease prevalence (82.8%) and medication use are high in the research group. This situation constitutes a significant cost for the healthcare system. Therefore, the presence of healthcare facilities where migrant elderly people can regularly monitor chronic diseases without experiencing communication problems and can easily access is important. In this way, both individually, the exposure of migrant elderly people to excessive disease burden can be prevented, and the economic cost imposed by Türkiye’s healthcare system can be reduced.

In old age, mental and social problems often occur as frequently as physical discomforts.^[52]^ It is of great importance for the health of the elderly to protect them from illness and disability, to perform their physical and cognitive functions, and to be socially productive.^[24]^ Functional assessment of daily living activities, which represents a functional approach in evaluating the health status of elderly individuals, is a method that provides important information.^[20]^ The vast majority of elderly immigrants in our research group (88.1%) can sustain their lives independently without support. Again, 86.1% of the participants were identified as not frail. This indicates that the dependency rate on clinical disease, daily living activities, exercise, and coping with illness is low. Conditions such as social, economic, and the presence of chronic diseases affect the ability of the elderly to perform daily living activities and their quality of life.^[21]^ Particularly, having the physical and cognitive capacity to sustain daily life independently in advanced age is of great importance as the ultimate health goal.^[7]^ It is noteworthy that the majority of immigrants in the research group can sustain their daily lives independently despite suffering from various chronic health problems.

Various studies have shown that various physical and mental health problems, along with sociodemographic characteristics, affect the daily living activities and frailty of the elderly.^[9,19,23,37,42,49]^ Various studies conducted in Chile, Spain, and Malaysia have shown that factors such as advanced age, low socioeconomic status, chronic disease, history of falls, and inadequate nutrition increase the frailty and dependency of the elderly.^[1,53,54]^ In our research group, factors such as age, education level, marital status, neurological problems, balance problems, history of falls, disability, chronic illness, and chronic pain were found to be associated with daily living activities and frailty. It is expected that younger, higher educated, married individuals living in crowded family environments without neurological and balance problems, history of falls, disability, and pain problems would have a higher rate of independently performing daily living activities. Because younger individuals are more physically active in their daily lives compared to older ones. The higher frequency of performing daily living activities among those with higher education levels may be associated with health literacy and the pursuit of health-preserving behaviors. In married individuals, the social and psychological support of the spouse may contribute to the more active daily lives of immigrants. Functional and neurological health problems are also factors that directly affect the ability of the elderly to perform daily activities. Advanced age, low education level, neurological and balance problems, disability, chronic pain, and chronic illness lead to increased dependence and frailty due to the physical weakening, decrease in cognitive and social capacities, and functional impairments of the elderly. Health literacy levels, cultural adaptation, and increasing social support mechanisms are important in reducing frailty in elderly immigrants.^[55]^

Nutrition is one of the most important components of health in the elderly and significantly affects the aging process. Factors such as decline in physical and mental functions, inadequate and unbalanced nutrition, and weakening of immunity increase the prevalence of malnutrition in the elderly.^[10]^ It has been shown that the prevalence of malnutrition in the elderly is between 13% to 28% in Türkiye and between 5% to 30% globally, reaching higher rates (up to 70%) in hospitalized and nursing home elderly individuals.^[56,57]^ In our study, the level of malnutrition (3.3%) among participants was very low, and the risk of malnutrition (20.5) was relatively high. This situation partly indicates that immigrant elderly individuals experience some problems with nutritional inadequacy. In a study conducted with immigrant elderly individuals in Iran, the prevalence of malnutrition was found to be 27%, and malnutrition was shown to be associated with depression and anxiety.^[58]^ Inadequate nutrition is widespread worldwide, posing a serious burden on both health facilities and societies.^[59]^ The presence of malnutrition increases hospitalization periods and mortality rates associated with diseases.^[2,60]^ In addition, inadequate nutrition negatively affects daily life, cognitive functions, and frailty status.^[61]^

It is thought that the lower prevalence of malnutrition among immigrant elderly individuals in our study may also be associated with the social support provided by the family environment in which they live. Moreover, the social support provided by both public and civil society organizations in Türkiye may contribute to this result. Indeed, in Türkiye, immigrants need to be officially registered to benefit from the health, education, and social support services provided by the public authorities. Syrian immigrants who are registered can benefit from these services free of charge with the identity card provided. In addition, many civil society organizations operating widely in Türkiye support Syrian immigrants in health, education, food, housing, and psychosocial services.^[62]^ These social support mechanisms, which respond to the various dimensions of immigrants’ needs, help immigrants integrate into society in Türkiye. However, the rate of 20.5% at risk of malnutrition deserves more detailed research. It can be assumed that the factors causing malnutrition risk may be related to inadequate and unbalanced nutrition due to low socioeconomic level or cultural incorrect eating habits. Therefore, comprehensive nutrition research in immigrant groups can reveal the factors leading to the emergence of malnutrition risk.

In our research group, advanced age, BMI ≤ 25, neurological and gait-balance problems, disability, chronic pain, and smoking status were found to be associated with malnutrition. Advanced age, neurological problems, disability, and chronic illness affect individuals’ ability to live independently, as well as their healthy and balanced nutrition, both physically and cognitively. It is known that low BMI poses a risk for malnutrition. In various studies conducted with the elderly, it has been shown that functional capacity decreases and there is a decrease in daily life activities in obese elderly individuals, which increases both the clinical frailty risk and the nutritional impact.^[3]^ The relationship between BMI and malnutrition is stronger in the presence of chronic diseases,^[63–65]^ and early detection of malnutrition is important both to reduce morbidity and mortality and to improve the quality of life of the elderly.^[66,67]^

The ability to perform daily living activities is associated with frailty and malnutrition. In our study, as the level of clinical frailty increased, the risk of malnutrition increased, and the level of independence of immigrant elderly individuals in performing daily living activities decreased. There are studies showing a positive relationship between malnutrition and clinical frailty.^[19,68]^ Improving the nutritional status of the elderly may play a significant role in preventing frailty.^[69]^ In a study conducted in Türkiye, it was shown that the clinical frailty status in the elderly is associated with cognitive impairment, depression, and inadequate nutrition.^[38]^ A systematic review conducted in Spain revealed that living alone, being unmarried, and having a low income level are associated with inadequate nutrition and malnutrition risk.^[70]^ The clinical frailty status of immigrant elderly individuals affects their ability to perform daily living activities and their malnutrition status. Identifying the risk factors associated with malnutrition in immigrant elderly individuals is important for improving their nutritional status.^[71]^

### 4.1. Strengths and limitations of the study

Türkiye is one of the countries that host the highest number of immigrants globally. Therefore, studies conducted in Türkiye regarding immigrants will make significant contributions to the literature. Furthermore, immigrant women, children, and the elderly constitute the most disadvantaged groups. Our study was conducted face-to-face with immigrant elderly individuals in their own homes in a district with a high immigrant population. This study, carried out with a hard-to-reach group in their own environments with real-time measurements, fills an important gap in the literature. On the other hand, conducting the research in only one district, the refusal of some immigrants to participate in the study, and therefore the limited number of participants, necessitate the consideration that the research findings may not be applicable to all immigrants in Türkiye. Additionally, caution is required regarding the generalizability of the research results.

## 5. Conclusion

According to the research findings, while 88.1% of immigrant elderly individuals are able to maintain their daily lives independently without relying on others, 13.9% are clinically fragile, and 23.8% are identified as being at risk of malnutrition or malnourished. The daily life activities, clinical fragility, and malnutrition status of immigrant elderly individuals have been associated with advanced age, low educational level, unmarried status, presence of neurological and balance problems, history of falls, disability, chronic illness, chronic pain, and BMI.

Enhancing the physical, cognitive, and social capacities of disadvantaged groups such as elderly immigrants can enable them to lead healthier daily lives independently and play a significant role in preventing nutrition-related issues. Therefore, the development of easily accessible social support programs tailored to immigrant groups will make a significant contribution.

Implementing an effective and sustainable malnutrition prevention/intervention program for elderly immigrants is of great importance. In this regard, conducting high-participation and multicenter field studies to assess the nutritional status of elderly immigrants and identify risk factors is crucial.

There is a need for comprehensive longitudinal studies that periodically follow individuals over time to identify and track the health status of immigrants in Türkiye and determine the health priorities of immigrant groups. This can contribute to the development of health policies tailored to specific age groups, particularly immigrant elderly individuals, based on their needs.

The Ministry of Health conducting periodic research to evaluate the healthcare services provided to Syrian immigrants and increasing the number of strengthened immigrant health centers with the inclusion of Syrian healthcare workers will facilitate immigrants’ access to healthcare services and make it easier to conduct costly chronic disease follow-ups within the healthcare system.

## Acknowledgments

We would like to express our gratitude to Dr Servet Yüce for his invaluable linguistic support.

## Author contributions

**Conceptualization:** Mehmet Sait Değer, Mehmet Akif Sezerol, Muhammed Atak.

**Data curation:** Mehmet Sait Değer, Mehmet Akif Sezerol, Muhammed Atak.

**Formal analysis:** Mehmet Sait Değer, Mehmet Akif Sezerol, Muhammed Atak.

**Funding acquisition:** Mehmet Sait Değer, Mehmet Akif Sezerol, Muhammed Atak.

**Investigation:** Mehmet Sait Değer, Mehmet Akif Sezerol, Muhammed Atak.

**Methodology:** Mehmet Sait Değer, Mehmet Akif Sezerol, Muhammed Atak.

**Project administration:** Mehmet Akif Sezerol.

**Resources:** Mehmet Sait Değer, Mehmet Akif Sezerol, Muhammed Atak.

**Software:** Mehmet Sait Değer, Mehmet Akif Sezerol, Muhammed Atak.

**Supervision:** Mehmet Sait Değer, Mehmet Akif Sezerol, Muhammed Atak.

**Validation:** Mehmet Sait Değer, Mehmet Akif Sezerol, Muhammed Atak.

**Visualization:** Mehmet Sait Değer, Mehmet Akif Sezerol, Muhammed Atak.

**Writing – original draft:** Mehmet Sait Değer, Mehmet Akif Sezerol, Muhammed Atak.

**Writing – review & editing:** Mehmet Sait Değer, Mehmet Akif Sezerol, Muhammed Atak.
